# Conformal Graphene-Decorated Nanofluidic Sensors Based on Surface Plasmons at Infrared Frequencies

**DOI:** 10.3390/s16060899

**Published:** 2016-06-16

**Authors:** Wei Wei, Jinpeng Nong, Linlong Tang, Guiwen Zhang, Jun Yang, Wei Luo

**Affiliations:** 1 Key Laboratory of Optoelectronic Technology & Systems, Ministry of Education of China, Chongqing University, Chongqing 400044, China; nongjp@cigit.ac.cn; 2 College of Optoelectronic Engineering, Chongqing University, Chongqing 400044, China; 20130813059@cqu.edu.cn (G.Z.); luo@cigit.ac.cn (W.L.); 3 Chongqing Institute of Green and Intelligent Technology, Chinese Academy of Sciences, Chongqing 401122, China; tll@cigit.ac.cn (L.T.); jyang@cigit.ac.cn (J.Y.); 4 Chongqing Engineering Research Center of Graphene Film Manufacturing, Chongqing 401329, China

**Keywords:** sensor, surface plasmons, nanofluidic, conformal grapheme, infrared

## Abstract

An all-in-one prism-free infrared sensor based on graphene surface plasmons is proposed for nanofluidic analysis. A conformal graphene-decorated nanofluidic sensor is employed to mimic the functions of a prism, sensing plate, and fluidic channel in the tradition setup. Simulation results show that the redshift of the resonant wavelength results in the improvement of sensitivity up to 4525 nm/RIU. To reshape the broadened spectral lines induced by the redshift of the resonant wavelength to be narrower and deeper, a reflection-type configuration is further introduced. By tuning the distance between the graphene and reflective layers, the figure of merit (FOM) of the device can be significantly improved and reaches a maximum value of 37.69 RIU^−1^, which is 2.6 times that of the former transmission-type configuration. Furthermore, the optimized sensor exhibits superior angle-insensitive property. Such a conformal graphene-decorated nanofluidic sensor offers a novel approach for graphene-based on-chip fluidic biosensing.

## 1. Introduction

Lab-on-chip systems combined with surface plasmon resonance (SPR) sensors provide a powerful technique to perform label-free biomolecular interaction measurements with high sensitivity [1,2,3]. A typical lab-on-chip system consists of a SPR excitation setup, a sensing plate, and a fluidic channel. In the traditional Kretschmann configuration [4], the prism used as the excitation setup is isolated from the sensing plate and the fluidic channel [5]. This means that the system needs to be attached and detached in each independent experiment, which largely decreases the efficiency and the usability. Furthermore, the prism needs to be angled appropriately to ensure the optimum output of incident light, hence making the system more complicated and less portable. To overcome these issues, a prism-free all-in-one setup is proposed, combining the SPR excitation item, sensing plate and flow channel in a single setup. For instance, Yang Hyun Joo *et al.* [6] demonstrated a long-range surface plasmon-polariton waveguide sensor employing an asymmetric double-electrode waveguide configuration. This consists of a microfluidic channel and a Bragg grating layer, which can effectively measure the refractive index of the inserted analyte. Ken-Ichi Nomura *et al.* [7] introduced a V-shaped trench-sensing system that minimizes the system and is unnecessary to adjust optical alignment, which contributes to the improvement of the detection efficiency. The above all-in-one setups extremely simplify the sensing system and reduce the sample volumes, promoting the development of the nanofluidic chip. However, these structures are based on the SPR in noble metals, of which the working wavelength is mainly located at the visible frequencies. While at mid-IR frequencies, these structures exhibit extremely large ohmic loss due to the low carrier mobility and increasingly large permittivity in magnitude [8]. Additionally, the metals cannot adsorb the biomolecules effectively because of the high surface inertness and intrinsic hydrophobicity [9]. Further extension of SPR sensors from visible and near-IR frequencies to mid-IR frequencies remains a significant challenge.

In contrast to the metals, graphene [10] that emerged as a unique two-dimensional carbon atoms possesses a high surface-to-volume ratio and strong π-π stacking interaction with the carbon-based ring structures in biomolecules [11]. Thereby, it can effectively adsorb the biomolecules on its surface. Particularly, graphene supports propagating surface plasmons with smaller loss, stronger confinement, and gate-tunability at the mid- and far-infrared frequencies [12,13]. These superior features render graphene a promising candidate for engineering infrared SPR sensors. Up to now, graphene-based SPR sensors have mainly employed the localized plasmons in patterned graphene [14,15] or surface plasmons in continuous graphene [16,17]. In these devices, the sample solution (fluidic biomolecules or liquid chemical reagent) would be dropped on the sensor surface for detection. As a consequence, the thickness of the sample solution cannot be effectively controlled, which introduces uncertainties in the measurement results. To alleviate this problem, an extra flow channel is added to contain the sample solution in order to accurately control the sample volume, as reported in [9]. However, this makes the system more complicated and hence lacks stability. Recently, a new type of sensor that integrates nanofluidic channel with a flat graphene sheet is reported [18]. In such a configuration, the nanofluidic channel can not only contain the sample solution, but also directly excite graphene surface plasmons (GSPs) without the need of an extra optical component, which significantly reduces the complexity of the device. However, the sensitivity of the sensor is limited (1920 nm/RIU with an optimal device) due to the small contact area between the sample solution and graphene.

Herein, we propose a novel infrared sensor based on graphene surface plasmons by decorating a conformal graphene [19,20,21] on a nanofluidic channel. Such a configuration allows for the detection of biological molecules in an aqueous environment. Besides, the conformal graphene can preserve the excellent electronic property of graphene and enlarge the contact area between graphene and biomolecules. A physical model was built employing the finite element method to improve the sensitivity of the sensor by optimizing the structure parameters of nanofluidic channels and Fermi level of graphene. Furthermore, a reflection-type configuration is proposed to improve the FOM while maintaining the high sensitivity.

## 2. Structure and Principles

**Figure 1:**
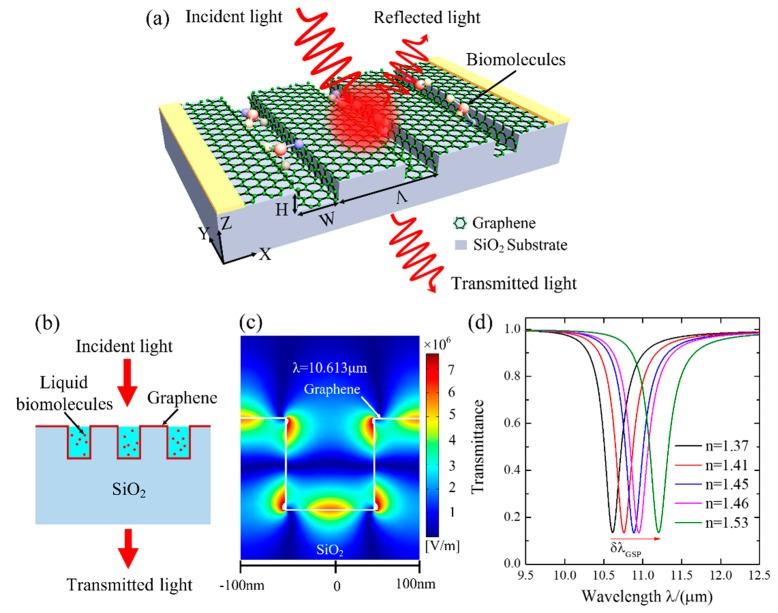
(**a**) Schematic of the conformal graphene-decorated nanofluidic channel (CGDNC) infrared sensor. (**b**) The cross section of the CGDNC. (**c**) The mode profiles of graphene plasmonics. (**d**) Transmission spectra when the refractive index of sensing medium is 1.37, 1.41, 1.45, 1.46, and 1.53, respectively (refractive indices of some typical molecules are in this range, such as double-stranded DNA [9]).

The structure of the proposed conformal graphene-decorated nanofluidic channel (CGDNC) infrared sensor is schematically illustrated in Figure 1a. A conformal graphene is decorated on an open nanofluidic channel array etched on a SiO_2_ substrate. In this case, the conformal graphene becomes hydrophilic due to the short-range chemical forces bonding between graphene and water [22]. Then, the sample fluidic can flow into the nanochannels for detection. The nanofluidic channels contain the following structural parameters: the period *Ʌ*, the width *W*, and the height *H*. A normally incident light with transverse magnetic polarization is used to excite the surface plasmon mode, and the transmitted light is detected as the signal of the change of the refractive index in the channels, as sketched in the cross section in Figure 1b.

Since the proposed open nanofluidic channels are periodic arrays, it also can be regarded as an optical gating. When the light irradiates from the top of the channel arrays, it will be scattered into evanescent waves with various diffraction orders due to the introduced periodic modulation originating from different regions of the channel [23]. Once the wave vector of an evanescence wave matches the dispersion relation of a GSP mode, the incident photons couple with the electrons on the surface of graphene, and a GSP wave can be excited [24]. Then, the incident light is absorbed strongly due to the excitation of the GSP wave, and a resonant dip is observed in the transmission spectrum. This resonant dip is very sensitive to the refractive index change in the channel induced by the adsorption of biomolecules at the graphene interface. The change in refractive index, δ*n_d_*, can be measured by detecting the wavelength shift, δλ*_GSP_*. The sensitivity S of a SPR sensor can be defined as the ratio of the wavelength shift to the change in refractive index

(1) $$S=\delta \lambda_{GSP}/\delta n_{d}$$

Equation (1) indicates that the sensitivity is decided by the resonant wavelength, and a large wavelength shift is highly desired, as it allows for the detection of a small refractive index change.

To obtain the resonant spectra of the sensor, a physical model is built based on the finite element method employing *Comsol mutiphysics*. In the model, the dielectric constant of the channel is 2.1 [25]. Graphene is modeled as a monolayer with a thickness of 0.34 nm. For the consider infrared frequency region where the photon energy *ħ*ω is much smaller than 2*E_f_*, the interband absorption of graphene is Pauli blocked and the surface conductivity of graphene can be simply characterized by the Drude model accounting only for intraband transition [26]:

(2) $$\sigma_{\mathrm{int}ra}\left(\omega \right)\approx \frac{e^{2}E_{f}}{\pi \hbar^{2}}\frac{i}{\omega +i\tau^{-1}}$$

where *e* is the elementary charge, *E_f_* is the Fermi level in graphene, τ is the relaxation time of charge carriers, ω is the angular frequency of incident light, and *ħ* is the reduced Planck’s constant.

The excited GSP mode when *Λ* = 200 nm, *W* = 100 nm, *H* = 100 nm, and *E_f_* = 0.3 eV is presented in Figure 1c. It is seen that the excitation of GSP exhibits a strong ability to couple the incident-free space light into the GSP wave and concentrate optical energy into sub-wavelength spots with a near-field |Ex| peak intensity of ~7.5 × 10^6^ V/m on the graphene surface. In addition, the optical energy of GSP wave is dissipated while propagating along the graphene due to the ohmic loss. Then, a dip can be observed in the transmission spectra at the resonant wavelength, λ*_GSP_*, as shown in Figure 1d. One can see that the resonant wavelength redshifts from 10.613 μm to 11.204 μm as the refractive index increases from 1.37 to 1.53, corresponding to a sensitivity of *S* = 3694 nm/RIU. To further improve the sensitivity, the structure parameters of the channel and the property of graphene are optimized in the following context.

## 3. Improvement of the Sensitivity

### 3.1. Effect of the Structure Parameters of CGDNC on the Sensitivity

**Figure 2:**
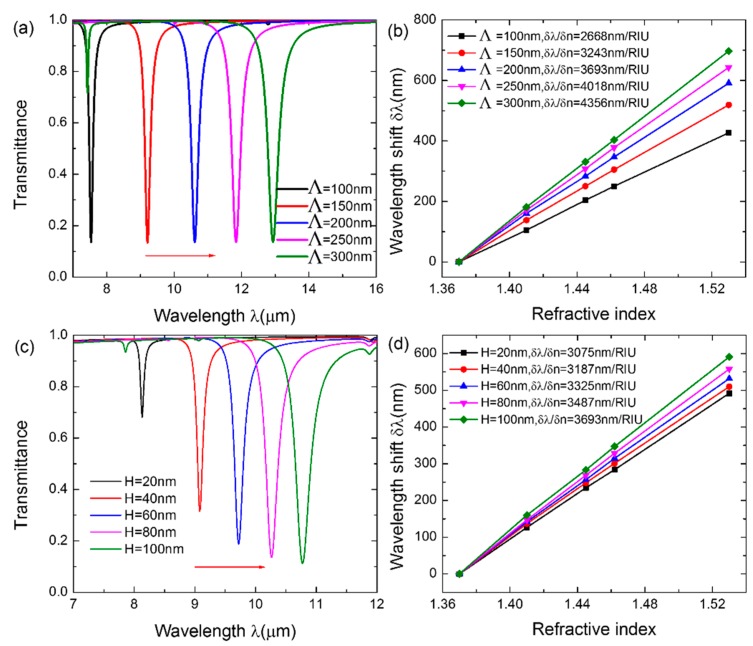
(**a**) Transmission spectra with varying *Λ* when *H* = 100 nm and *W* = 100 nm. (**b**) The calculated sensitivity when *Λ* = 100 nm, 150 nm, 200 nm, 250 nm, and 300 nm, respectively. (**c**) Transmission spectra with varying H when *Λ* = 200 nm and *W* = 100 nm. (**d**) The calculated sensitivity when *H* = 20 nm, 40 nm, 60 nm, 80 nm, and 100 nm, respectively. The lines in (**b**) and (**d**) are linear fittings to the data.

Simulation results show that the spectral characteristics and the performance of the sensor can be modulated by tuning the period *Λ* and height *H* of the CGDNC. The transmission spectra with varying *Λ* when *H* = *W* = 0.5*Λ* is illustrated in Figure 2a. One can see that the resonant wavelength redshifts from 7.535 μm to 12.935 μm as *Λ* increases from 100 nm to 300 nm. To evaluate the sensing performance of the sensor, the resonant wavelength shift as a function of refractive index is plotted in Figure 2b. The linear fitting results indicate that the sensitivity of the sensor is significantly improved by 63.3% from 2668 nm/RIU to 4356 nm/RIU. Figure 2c presents transmission spectra with varying *H* when *Λ* = 200 nm and *W* = 100 nm. It shows that the resonant wavelength redshifts from 8.134 μm to 10.773 μm as *H* increases from 20 nm to 100 nm. Additionally, as plotted in Figure 2d, the corresponding sensitivity improves from 3075 nm/RIU to 3693 nm/RIU. This is attributed to the redshift of the resonant wavelength and, partly, the immense increase of the contact area between graphene and biomolecules. Therefore, the sensitivity of the sensor can be improved by increasing the period and height of the CGDNC in the initial design.

### 3.2. Effect of the Coupling of GSP Modes on the Sensitivity

**Figure 3:**
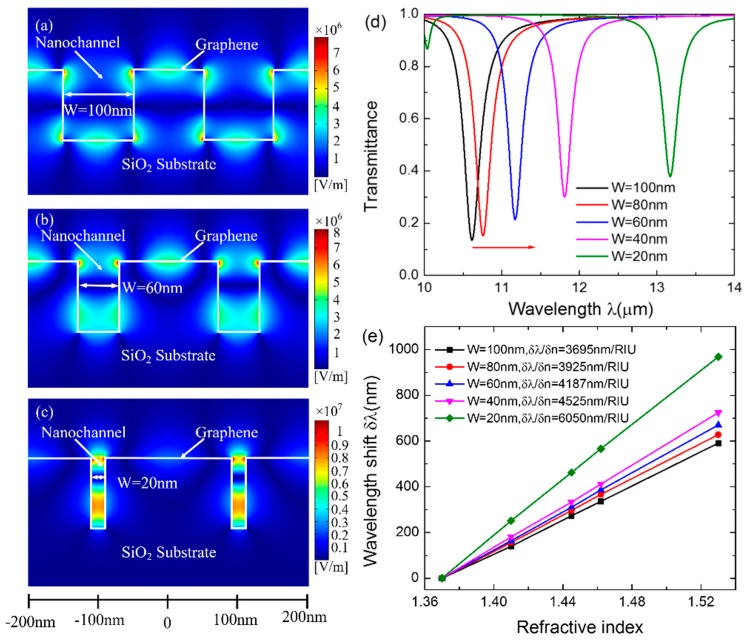
The GSP mode profiles when (**a**) *W* = 100 nm, (**b**) 60 nm, and (**c**) 20 nm in two periods. (**d**) Transmission spectra with decreasing *W* from 100 nm to 20 nm when *Λ* = 200 nm and *H* = 100 nm. (**e**) The calculated sensitivity when *W* = 100 nm, 80 nm, 60 nm, 40 nm, and 20 nm, respectively. The lines are linear fittings to the data.

Further investigation indicates that the GSP modes propagating along the inner sides of the channel strongly couple together as the width *W* of the channel decreases, resulting in the significant improvement of the sensitivity. The corresponding GSP mode profiles in two periods when *W* = 100 nm, 60 nm and 20 nm are shown in the Figure 3a,b,c, respectively. As expected, when *W* = 100 nm (Figure 3a), the GSP modes propagating along the inner sides of the channel are isolated from each other. Then, the adjacent GSP modes in the channel couple with each other when *W* decreases to 60 nm (Figure 3b). As *W* further decreases to 20 nm, the GSP modes completely overlap and couple strongly, forming a hybrid coupled mode at the bottom and the ridge edges of the channel (Figure 3c). The strong coupling of GSP modes significantly enhance the peak intensity of the localized electric field by 39.7% in the channel and results in the redshift of the resonant wavelength by 24.1% from 10.613 μm to 13.171 μm, as shown in Figure 3d. Such behavior extremely enhances the interaction between the fluidic biomolecules and the incident light and makes it more sensitive to the change in the refractive index, which contributes to the improvement of sensitivity. As indicated in Figure 3e, the sensitivity increases by 63.1% from 3695 nm/RIU up to 6025 nm/RIU as *W* decreases from 100 nm to 20 nm. Thus, in order to gain a higher sensitivity of the sensor, a narrower channel should be designed to ensure the strong coupling of GSP modes along the inner sides of the channel. Considering the processing craft of the nanofluidic channel and the incorporation of the channel with graphene, a width of 40 nm was chosen for the following analyses.

### 3.3. Effect of the FERMI Level of Graphene on the Sensitivity

**Figure 4:**
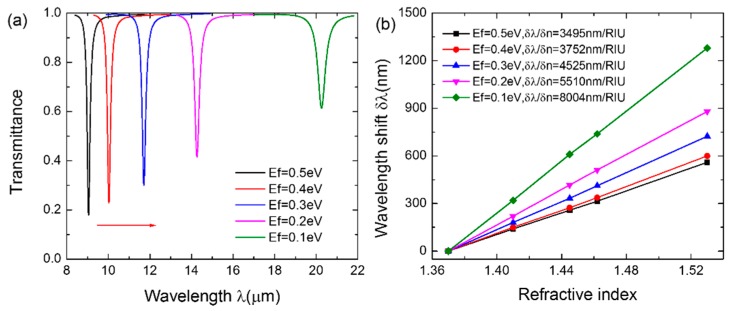
(**a**) The transmission spectra with varying *E_f_* when *Λ* = 200 nm, *H* = 100 nm, and *W* = 40 nm. (**b**) The calculated sensitivity when *E_f_* = 0.5 eV, 0.4 eV, 0.3 eV, 0.2 eV, and 0.1 eV, respectively. The lines are linear fittings to the data.

Since the structure parameters of CGDNC can be only tuned during the fabrication, they cannot be dynamically controlled after the fabrication of the sensor. Therefore, we further considered actively improving the sensitivity by adjusting the Fermi level *E_f_* of graphene. The evolution of the transmission spectra with varying *E_f_* is illustrated in Figure 4a. The figure indicates that the sensor can operate within a wide range of infrared wavebands by applying an external gate voltage. The resonant wavelength redshifts from 9.15 μm to 20.45 μm as *E_f_* decreases from 0.5 eV to 0.1 eV. This results in the improvement of the sensitivity from 3495 nm/RIU to 8004 nm/RIU, as shown in Figure 4b. Consequently, the sensitivity of the sensor can be actively improved by gate-tuning the external voltage to decrease the Fermi energy level of graphene after the fabrication of the device.

## 4. Improvement of the FOM

**Figure 5:**
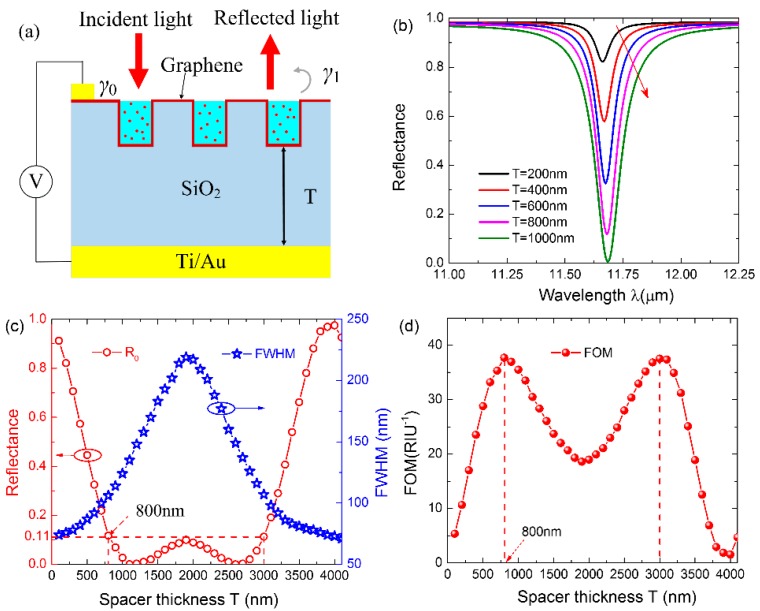
(**a**) Schematic of the reflection-type CGDNC infrared sensor; (**b**) The reflection spectra with varying spacer thickness *T* when *Λ* = 200 nm, *H* = 100 nm, *W* = 40 nm, and *E_f_* = 0.3 eV; (**c**) The extracted R_0_ and fwhm from the reflection spectra with varying spacer thickness; (**d**) The calculated FOM with varying spacer thickness.

The above analysis brings us to the conclusion that the redshift of the resonant wavelength can significantly improve the sensitivity of the sensor by optimizing the structural parameters of CGDNC and the Fermi level of graphene. However, the redshift of the resonant wavelength of such a transmission-type sensor is always accompanied with the broadening of the resonant dip (shown in Figure 2a,c, Figure 3d and Figure 4a). This results in the decrease in detection accuracy (defined as the reciprocal of the full width at half maximum (fwhm)). This means that a higher sensitivity is generally at the cost of a lower detection accuracy and that there is a trade-off between sensitivity and detection accuracy. To effectively evaluate the overall performance of sensor, the figure of merit (FOM) is defined as in [27,28]:

(3) $$FOM=S\frac{\Delta R}{fwhm}$$

where ΔR is the resonance depth, and S is the sensitivity. Since the sensitivity is closely related to the resonant wavelength, the key to improve the detection accuracy is to reshape the spectral line to have a large resonance depth and a small fwhm while keeping the resonant wavelength unchanged. To this end, we further propose a reflection-type sensor with an Au layer at the bottom of the SiO_2_ layer, as sketched in Figure 5a. A 5-nm Ti layer is sandwiched between the Au layer and the SiO_2_ layer in order to improve their adhesion. In such a configuration, an asymmetric Fabry–Perot (F–P) cavity is formed between the graphene and reflective layers, which allows the incident light to make two passes (forward and reflected) through the graphene sheet. The spectral line can be reshaped to modulate the graphene–light interaction by adjusting the F–P cavity length. The optimized parameters *Λ* = 200 nm, *H* = 100 nm, *W* = 40 nm, and *E_f_* = 0.3 eV (corresponding to *S* = 4525 nm/RIU) are used in the following analysis.

Simulation results indicate that the FOM of the sensor can be modulated periodically by tuning the F–P cavity length (spacer thickness *T*). The reflection spectra with several sets of spacer thickness *T* are presented in Figure 5b. As expected, the notch broadens and becomes deeper as *T* increases from 200 nm to 1000 nm. To quantitatively reveal the reshaping of the spectral line, the *R_a_* and fwhm are further extracted from the reflection spectra in a wide range of *T* from 100 nm to 4100 nm and plotted in Figure 5c. This suggests that *R*_0_ and fwhm vary periodically with *T*, and this results in the periodical variation of FOM with the same period of 4000 nm, as plotted in Figure 5d. The FOM obtains two maximum value of 37.69 RIU^−1^ in a period. According to the F–P physical model, the variation period of the FOM is determined by the resonant wavelength λ_0_ and refractive index *n* of the spacer through *T*_0_ = λ_0_/2*n*, which is coincident with the results in Figure 5d. Therefore, the FOM can achieve a maximum value periodically by fabricating a suitable F–P cavity length in the initial design.

To further quantitatively reveal the condition to obtain the maximum FOM, the sensor is modeled as a resonator with a resonant frequency of ω_0_ and an intrinsic loss rate of γ_0_, which couples with the incident TM wave with a leakage rate of γ_1_ (Figure 5a). According to the temporal coupled mode equations [15,29], the reflectance of the resonator at ω can be given by

(4) $$R=\frac{{\left(\omega -\omega_{0}\right)}^{2}+{\left(\gamma_{0}-\gamma_{1}\right)}^{2}}{{\left(\omega -\omega_{0}\right)}^{2}+{\left(\gamma_{0}+\gamma_{1}\right)}^{2}}$$

This gives us two essential parameters (*R*_0_ and fwhm) of the spectral lines. The resonant reflectance *R*_0_ at ω *=* ω_0_ is represented as

(5) $$R_{0}=\frac{{\left(\gamma_{0}-\gamma_{1}\right)}^{2}}{{\left(\gamma_{0}+\gamma_{1}\right)}^{2}}$$

and the corresponding fwhm as

(6) $$fwhm=2(\gamma_{0}+\gamma_{1})$$

Equations (5) and (6) manifest that the intrinsic loss rate γ_0_ and the leakage rate γ_1_ are the key physical parameters that dominate the shape of the spectral line. Here, the intrinsic loss rate γ_0_ is the intrinsic property of graphene, which cannot be changed, while the leakage rate γ_1_ is determined by the spacer thickness *T*. Considering these two equations, the FOM can be further written as

(7) $$FOM=2S\frac{\gamma_{0}\gamma_{1}}{{\left(\gamma_{0}+\gamma_{1}\right)}^{3}}$$

According to the first-order derivative of Equation (7), there is a solution for obtaining the maximum FOM, *i.e.*, γ_1_ = 2γ_0_ for a fixed γ_0_. By substituting this solution into Equation (5), we learn that the FOM achieves the maximum value when reflectance *R*_0_ = 1/9, which agrees well with the simulation results in Figure 5c,d. Hence, a maximum FOM can be achieved by adjusting the F–P cavity length to satisfy the condition of *R*_0_ ≈ 11% in the initial design.

**Figure 6:**
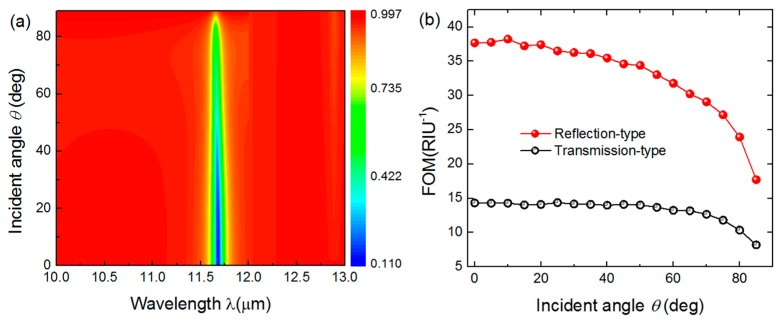
(**a**) Reflectance of reflection-type sensor mapping with varying incident angles and wavelength when *Ʌ* = 400 nm, *H* = 100 nm, *W* = 40 nm, and *E_f_* = 0.3 eV; (**b**) The calculated *FOM* of the transmission-type (black hollow sphere) and reflection-type (red solid sphere) sensors as a function of θ.

Finally, it is found that the proposed sensor can work in a wide angle range of incident light. The reflectance of the reflection-type sensor as a function of incident angles and wavelength when *T* = 800 nm is mapped in Figure 6a. One noticeable feature is that the narrow bandwidth and large resonance depth of the reflection spectrum are maintained until the incident angle is larger than 60°. This indicates that the proposed sensor possesses excellent angle-insensitive property, which is attributed to the deep sub-wavelength nature of graphene plasmons and the effects of Bragg scattering at the Brillouin zone center [16]. Such a feature contributes to the superior sensing performance of the device in a wide angle range, as plotted in Figure 6b. The FOM is maintained at a high level and reaches a maximum value of 37.69 RIU^−1^. Furthermore, the FOM of the reflection-type sensor is compared with that of the transmission-type in Figure 6b. As expected, the former one is 2.6 times larger than the latter one, signifying that the reflection-type structure gains an advantage over the transmission-type one from a practical point of view.

## 5. Conclusions

As a summary, a novel CGDCN sensor based on GSP is proposed. It is observed that the redshift of the resonant wavelength can significantly improve the sensitivity to a high sensitivity of 4525 nm/RIU by optimizing the structure parameters of CGDNC and the Fermi level of graphene. A reflection-type sensor is further proposed to improve the FOM of the sensor by reshaping the spectral line. A maximum FOM of 37.69 RIU^−1^ can be achieved by adjusting the F–P cavity length, which is 2.6 times larger than that of the transmission-type sensor. Moreover, the proposed sensor can work at a broad range of incident angles. Such a CGDNC sensor provides a green platform for on-chip biochemical fluidic analysis and finds potential applications in fields such as pharmaceuticals, environmental monitoring, and food safety.
